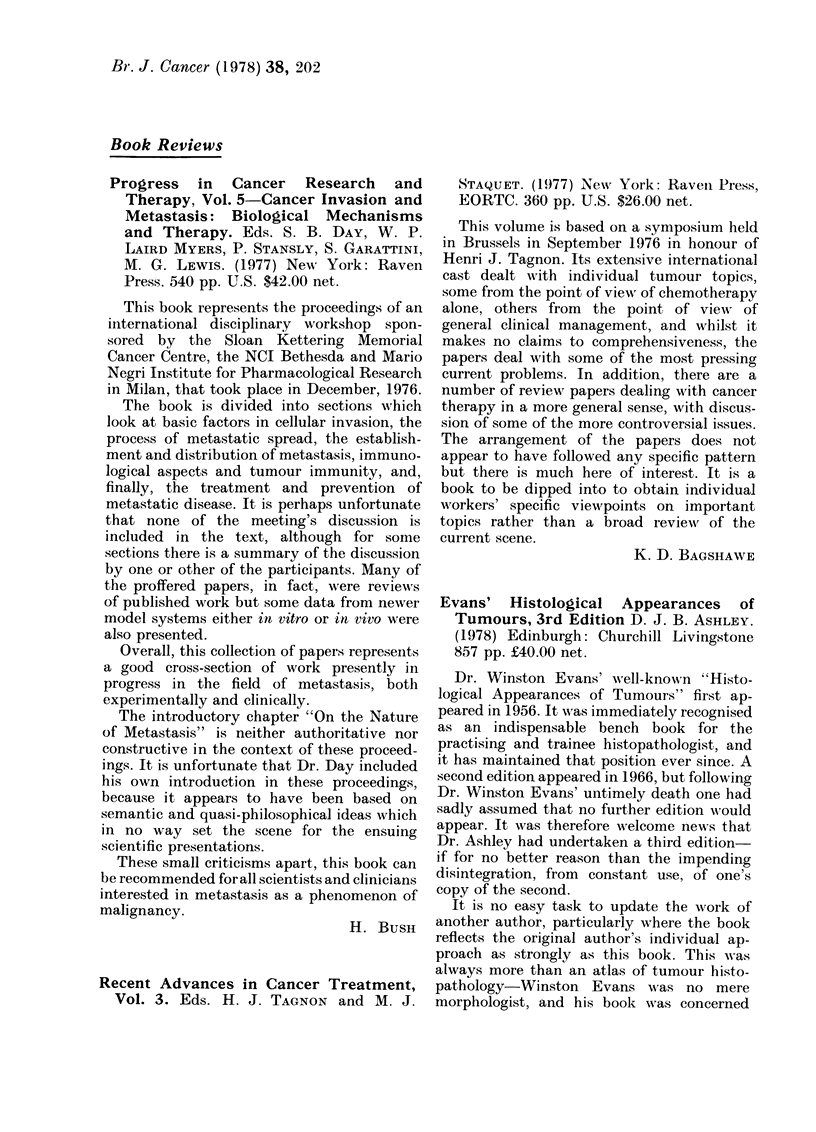# Recent Advances in Cancer Treatment, Vol. 3

**Published:** 1978-07

**Authors:** K. D. Bagshawe


**Recent Advances in Cancer Treatment, Vol. 3.** Eds. H. J. Tagnon and M. J. Staquet. (1977) New York: Raven Press, EORTC. 360 pp. U.S. $26.00 net.

This volume is based on a symposium held in Brussels in September 1976 in honour of Henri J. Tagnon. Its extensive international cast dealt with individual tumour topics, some from the point of view of chemotherapy alone, others from the point of view of general clinical management, and whilst it makes no claims to comprehensiveness, the papers deal with some of the most pressing current problems. In addition, there are a number of review papers dealing with cancer therapy in a more general sense, with discussion of some of the more controversial issues. The arrangement of the papers does not appear to have followed any specific pattern but there is much here of interest. It is a book to be dipped into to obtain individual workers' specific viewpoints on important topics rather than a broad review of the current scene.

K. D. Bagshawe